# Supplementary material for: Using Dried Blood Spots for a Sero-Surveillance Study of Maternally Derived Antibody against Group B Streptococcus
Source: Vaccines (Basel). 2023 Feb 4;11(2):357. doi: 10.3390/vaccines11020357 (PMC9966576; doi:10.3390/vaccines11020357)
Supplement: Supplementary file 1 [file vaccines-11-00357-s001.zip › vaccines-2127585-supplementary.pdf]

Supplementary Material

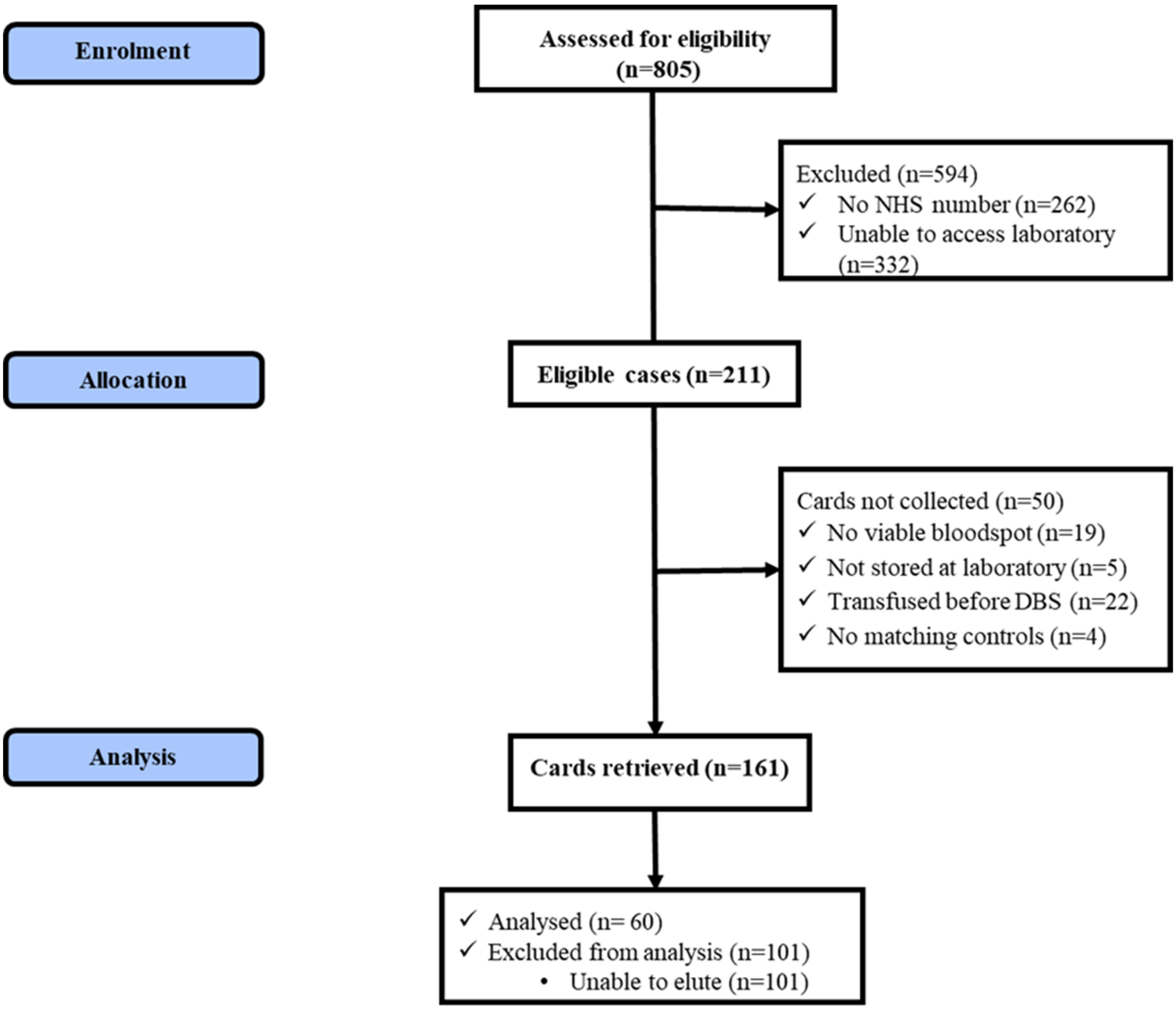

Figure S1. Consort diagram of GBS cases DBS card sample collection.

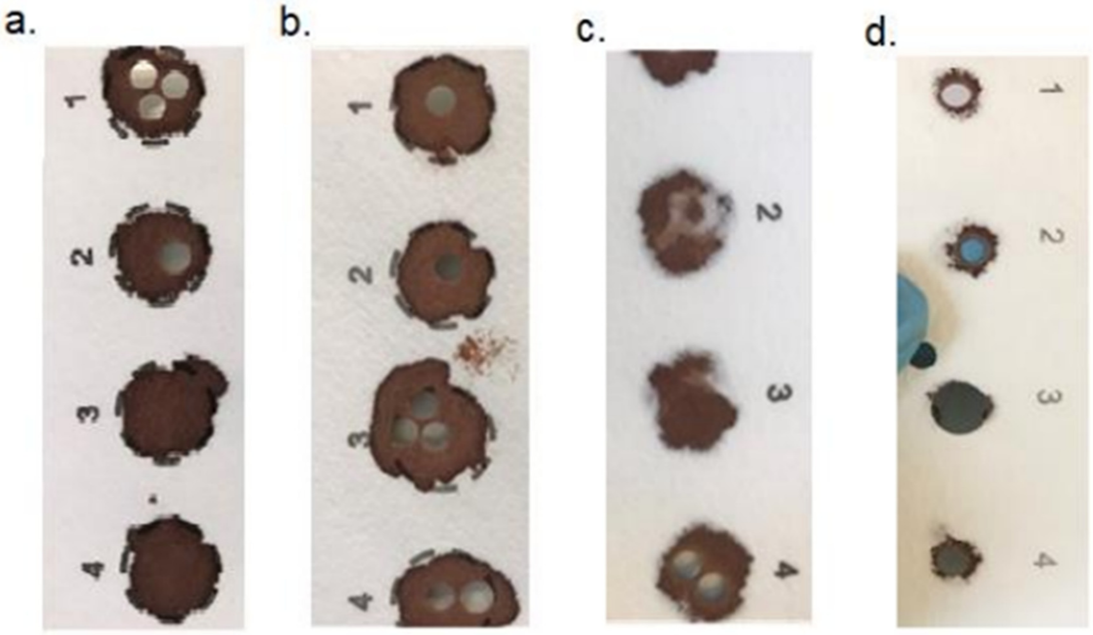

Figure S2. Viable and non-viable blood spots for collection (a. DBS 1 is inadequate, 2 is adequate, 3 and 4 are ideal. b. DBS 1 and 2 are adequate but not ideal. DBS 3 and 4 are not viable. c. DBSs are not viable as blood has not soaked through the card. d. DBS already punched for laboratory testing and there no blood is left).
